# ASSESSING AND STRENGTHENING THE INFRASTRUCTURE FOR CAREGIVING SUPPORTS: A STATE PERSPECTIVE

**DOI:** 10.1093/geroni/igae098.2147

**Published:** 2024-12-31

**Authors:** Jennifer Rabalais, Alison Rataj

**Affiliations:** University of New Hampshire, Concord, New Hampshire, United States; University of Massachusetts Boston, Durham, New Hampshire, United States

## Abstract

Objective. While progress has been made in policies that recognize and support caregivers, there is a need for a unified framework for caregiver supports. The New Hampshire Alliance for Healthy Aging (NHAHA), a statewide coalition focused on building meaningful partnerships to support and promote healthy aging in communities, has a focal area of ensuring that informal family caregivers have adequate supports. Through NHAHA, we examined current state data, policies, and practices that could be leveraged to develop a recommended plan of actions for caregiving supports that aligns with national caregiving strategies. Methods. Using a mixed-methods design, this study used qualitative data from a focus group (n = 14) to assess barriers to accessing caregiving services. A statewide survey (2024) is underway to understand existing services and supports in NH and will be used in further analysis. Data from NH’s State Plan on Aging (2023), which conducted a survey (n = 955) asking several questions about caregiving supports and hosted 10 public listening sessions (n = 185) was also used. Data was catalogued and inventoried across themes in this applied research approach. Results. Three major themes emerged. Caregivers often do not self-identify. Caregiving has negative effects on caregivers financial standing and career. Caregiving supports are siloed, difficult to navigate, and often dissolve without warning. Future Directions. Increased education for caregivers that provides information and resources. Create a system of care for caregivers including respite and financial relief. Advance caregiver friendly work environments.

